# Mechanism and Performance of Bituminous Mixture Using 100% Content RAP with Bio-Rejuvenated Additive (BRA)

**DOI:** 10.3390/ma15030723

**Published:** 2022-01-18

**Authors:** Anqi Chen, Yazhen Qiu, Xiangyu Wang, Yuanyuan Li, Shaopeng Wu, Quantao Liu, Fan Wu, Jianlin Feng, Zhuowei Lin

**Affiliations:** 1 Wuhan Institute of Technology, School of Civil Engineering and Architecture, Wuhan 430205, China; anqi.chen@nottingham.ac.uk (A.C.); qiuyazhen111412@163.com (Y.Q.); wxy20010407@163.com (X.W.); 22004010123@stu.wit.edu.cn (F.W.); 22004010110@stu.wit.edu.cn (J.F.); 2 Nottingham Transportation Engineering Centre, School of Civil Engineering, University of Nottingham, University Park, Nottingham NG7 2RD, UK; 3 State Key Laboratory of Silicate Materials for Architectures, Wuhan University of Technology, Wuhan 430070, China; wusp@whut.edu.cn (S.W.); liuqt@whut.edu.cn (Q.L.); 4 School of Literatures, Languages and Cultures, The University of Edinburgh, Edinburgh EH8 9LH, UK; S2249510@ed.ac.uk

**Keywords:** bio-rejuvenated additive, 100% rejuvenation, reclaimed asphalt pavement, regeneration mechanism, pavement performance

## Abstract

The low RAP content, hot mixing conditions, and the addition of a high ratio of new bitumen and aggregates result in low economic and environmental benefits for current regeneration technologies. A bio-rejuvenated additive (BRA) that can fully (100%) regenerate the RAP without heating is proposed in this paper. To reveal the mechanisms of BRA-rejuvenated RAP, the effects of BRA on the chemical structure and molecular weight of the RAP were investigated using Fourier-transform infrared spectroscopy and gel permeation chromatography. The mechanical performance and water damage resistance of BRA-rejuvenated RAP were studied. Low contents of new bitumen or epoxy resin were suggested to increase the mechanical performance of 100% RAP. The results show that the 1.5% BRA-rejuvenated RAP had the best mechanical performance. The blending of BRA with recycled RAP is a completely physical process, without any chemical reactions. The molecular weight of BRA is lower than that of bitumen; it can substantially increase the content of light components in aged bitumen, and play the role of adjusting and restoring the balance of the components of aged bitumen. The mechanical performance of BRA-rejuvenated RAP is enhanced significantly by adding low dosages of new bitumen or epoxy resin.

## 1. Introduction

Due to the good viscoelastic properties of bitumen, asphalt pavements are used as the main form of pavement structure worldwide [1]. Bituminous mixtures consist of aggregates, fillers, and organic bituminous binder. The bulk bituminous mixture can build strength after being paved and compacted on the road [2]. During service, the organic bituminous binder is aged by various effects, including environmental factors and vehicle loadings [3], leading to a gradual degradation of pavement performance and the occurrence of stresses in roads, such as cracks, spalling, ravelling, and potholes [4]. Typically, when the pavement condition index (PCI) of a bituminous pavement is lower than 75, the pavement must be repaired (or partially repaired) or reconstructed, resulting in a significant waste of bituminous mixtures [5]. Almost 35% of asphalt pavements in China needed to be repaired from 2016 to 2020, which produced more than 3.4 billion tons of reclaimed bituminous mixture (RAP), causing significant environmental harm and resource waste without being effectively regenerated. The construction of bituminous pavement requires large amounts of petroleum bitumen and aggregate. In 2020, China’s petroleum bitumen production was 62,796,000 tons, with approximately 85% applied to roads and airport runways, and the bitumen and aggregate consumption was estimated to be 53,377,000 tons and 1.07 billion tons, respectively [6,7]. Therefore, the recycling of RAP is of great significance, as it can not only reduce the consumption of petroleum resources, but also reduce the cost of bituminous concrete.

In recent years, the research on RAP has gained more and more attention. Yu [8,9] investigated the fracture properties of the mixtures containing up to 40% RAP with polymer-modified bituminous (PMA) binder, and the results indicated that RAP mixtures with PMA exhibited lower damage accumulation rates than common RAP mixtures. The study of Juliana [8,9] to determine the variability of RAP properties obtained from different projects or sources indicated that there is significant variability in the performance of the RAP. Han [10] studied the impact of RAP on the pavement performance of bituminous mixtures. The findings indicated that the low-temperature performance and fatigue resistance decrease with the increase in RAP content; meanwhile, the water damage resistance of bituminous mixture first increases (with 0–40% RAP), and then decreases (>40% RAP). Wu [11] indicated that the bituminous mixture containing 40% RAP had the greatest shear strength. The RAP increases the potential for low-temperature cracking in bituminous mixtures [12]. Therefore, the RAP and its dosage have a significant influence on the pavement performance [10,13,14,15] and workability [16,17] of bituminous mixtures. To ensure that the bituminous mixtures containing RAP have good pavement performance [18], the “Technical Specifications for Highway Asphalt Pavement Recycling” in China indicate that the optimal RAP content should be between 15% and 30%, and most uses in actual engineering also do not exceed 30% [19,20]. Currently, both hot and cold recycled RAP technologies require the addition of new materials, such as new bitumen and aggregates [21,22], and the hot mixing process requires heating of both the new materials and the RAP (old and new aggregates are heated separately, with the new aggregates being heated to a higher temperature and acting as a secondary heat source to heat the RAP). These requirements result in high energy consumption [23,24] and low-content RAP recycling [25]. To increase the used content of RAP in bituminous mixtures, it is essential to stimulate and restore the technical properties of the aged bitumen covering the surface of the RAP [26]. The cold mixing technology can reduce the energy consumption and the elimination of lightweight components during heating, which can prevent acceleration of the aging of bitumen, and also reduce the volatility of volatile organic compounds (VOCs).

A bio-rejuvenated additive (BRA) for regenerating 100% RAP with cold mixing technology is proposed in this research. After mixing the BRA with RAP, the BRA can stimulate the aged bitumen, which is coated in a thin layer on the surface of the RAP aggregate, and restores its components and technical properties. To enhance the strength and toughness of BRA-rejuvenated RAP [27], low doses of new bitumen or epoxy resin were added to the 100% cold mixing of RAP with BRA to create a spatial three-dimensional network. Fourier-transform infrared spectrometer (FTIR) and gel permeation chromatography (GPC) were used to determine the chemical structure and molecular weight distribution of BRA-rejuvenated bitumen; the results can reveal the regeneration mechanism of BRA-rejuvenated RAP. In addition, the high-temperature performance, low-temperature performance, water damage resistance, and fatigue resistance of BRA-rejuvenated RAP were investigated extensively to evaluate the pavement performance of BRA-rejuvenated RAP.

## 2. Materials and Experimental Methods

### 2.1. BRA

The BRA was produced from the waste residues during the production of cooking oil, and is a sustainable regenerative agent for aged bitumen; it was supplied by the Wuhan Daosheng Transportation Technology Co., LTD, Wuhan, China. The technical parameters of the BRA are shown in Table 1.

### 2.2. Mix Design of BRA-Rejuvenated RAP

The RAP was crushed and divided into four grades of 10–15 mm, 5–10 mm, 3–5 mm, and 0–3 mm; the size distribution of each grade of RAP is shown in Table 2. The bitumen contents of different sizes of RAP aggregates are shown in Table 3. The actual bitumen content of the RAP mix was 4.5%.

The low dosages of new bitumen and epoxy resin were applied as the enhancement additives, in an effort to improve the mechanical properties and road performance of the BRA-rejuvenated RAP. The dosages of new bitumen and epoxy resin are shown in Table 4. The ratio of additives was measured by the weight of the RAP.

### 2.3. Chemical Structure Analysis Tests

The Fourier-transform infrared spectrometer (*FTIR,* NICOLET 6700 FLEX, Thermo Fisher, Waltham, MA, USA) was used to determine the chemical structure of unaged bitumen, aged asphalt, BRA, and BRA-rejuvenated RAP. The testing specimens were prepared at a ratio of 1:20 with carbon disulphide (CS_2_) solution according to the film method in GB/T 6040-2019, pressed into shape, and then subjected to FTIR spectroscopy. The scanning spectral wavenumber range of the test instrument was from 4000 cm^−1^ to 400 cm^−1^.

### 2.4. Molecular Weight Distribution Tests

Gel permeation chromatography (GPC, Agilent PL-GPC50, Varian, PaloAlto, Santa Clara, CA, USA) was used to measure the molecular weight distribution of the BRA. Firstly, 0.4 mg of BRA and 2 mL of tetrahydrofuran solution were placed in the bottle; then, the sample solution was filtered into the test glass container using a filter with a 0.45 μm pore size microporous filter membrane, and then 50 μL of the sample was injected into the injector. The flow rate of the solution through the gel column was 1 mL/min.

### 2.5. Mixing and Indoor Regeneration of BRA-Rejuvenated RAP

The different grades of RAP were mixed according to the mix design of the BRA-rejuvenated RAP, and then the BRA was added to the mixing machine and mixed for 120 s. The mixing temperature was atmospheric temperature. Then, the mixture was stored at atmospheric temperature for 24 h to allow the aged bitumen to be fully activated by the BRA. After 24 h, the epoxy resin was added to the BRA-rejuvenated RAP for a second mixing, with a mixing time of 90 s. The mixture was then used to manufacture the test specimens. The Marshall specimens of BRA-rejuvenated RAP were mixed in the same way as commonly used dense-grade HMA, and the specimens were compacted 75 times for each side. The specimens were cured at 40 °C for 2 days. For the BRA-rejuvenated RAP with enhancement additives (new bitumen or epoxy resin), firstly, the new bitumen was heated to 130 °C, and then the BRA was added to the melted bitumen; we then used a high-speed shearing machine to stir the mixture for 15 min. The BRA-modified bitumen was manufactured to regenerate the RAP. The low viscosity of the BRA-modified bitumen ensured that it could mix freely with the RAP at room temperature.

### 2.6. Asphalt Mixture Tests

#### 2.6.1. Water Damage Resistance Test

The Marshall test and splitting test were used to study the water damage resistance of the mixture; the loading rate was 50 mm/min. For the Marshall test, the unconditioned and the conditional Marshall specimens were held in a 25 °C water bath for 0 and 24 h, respectively; for the splitting test, the unconditioned specimens were held in a 15 °C water bath for 2 h, while the conditional specimens were first held in a 25 °C water bath for 22 h, and then in a 15 °C water bath for 2 h.

#### 2.6.2. Low-Temperature Cracking Resistance Test

The −10 °C semi-circular bending test was used to study the low-temperature cracking resistance of the mixture, using a universal testing machine (UTM-100, IPC Global, Alpharetta, GA, USA). This test was conducted according to the criterion of ASSHTO TP 105-13. The Marshall specimen (φ101.6 mm × 63.5 mm) was cut into two semicircles, and then a 5 mm deep and 3 mm wide groove was cut at the midpoint of the specimen. The specimen was conditioned at −10 °C for 2 h, at a loading rate of 0.5 mm/min.

#### 2.6.3. Fatigue Resistance Test

The fatigue resistance test of the mixture was carried out using a semi-circular bending test; the test was conducted using a universal testing machine ((UTM-100, IPC Global, Alpharetta, GA, USA)). The specimens of the fatigue test were manufactured according to ASSHTO TP 105-13. The stress ratios were 0.3, 0.4, 0.5, 0.6, 0.7, and 0.8. The specimens were conditioned at 25 °C for 6 h; the loading rate was 0.5 mm/min.

#### 2.6.4. Rutting Resistance Test

The rutting resistance of BRA-rejuvenated RAP was investigated with the wheel tracking test. The size of the rutting resistance test was 300 mm × 300 mm × 50 mm. Before the test, the specimens were conditioned at 60 °C for 6 h. The contact pressure of the loading wheel and the specimen surface was 0.7 MPa, and the rolling speed was 42 times/min. The dynamic stability was calculated by taking the test data from 45 min to 60 min.

### 2.7. Technical Map

**Figure 1:**
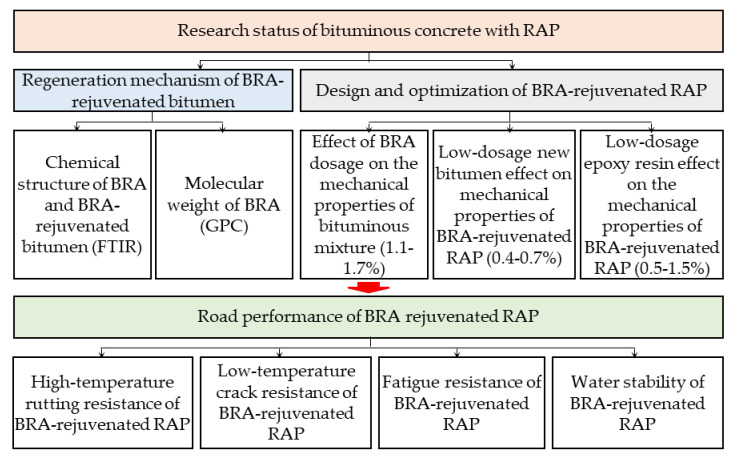
Technical map. (Where, BRA: Bio-rejuvenated additive; RAP: Reclaimed bituminous mixture; GPC: gel permeation chromatography; FTIR: Fourier transform infrared spectrometer.).

The Technical map of this research is shown in Figure 1.

## 3. Results and Discussions

### 3.1. Regeneration Mechanism of BRA-Rejuvenated RAP

#### 3.1.1. Molecular Weight of BRA

**Figure 2:**
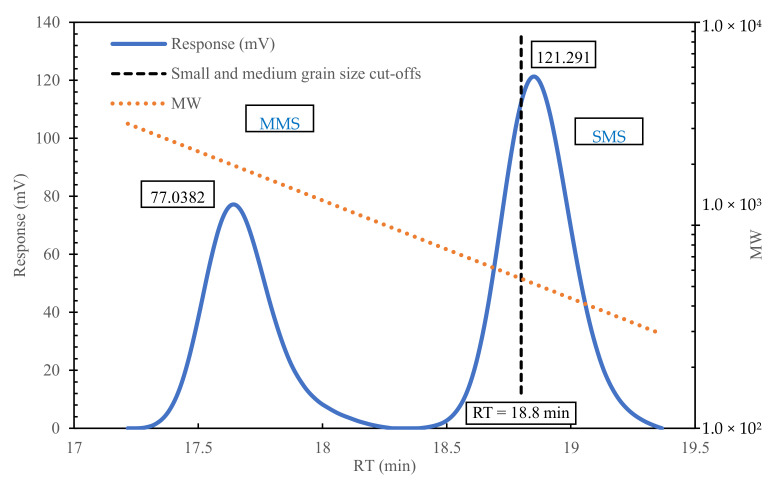
Gel permeation chromatography (GPC) analysis diagram of BRA.

The microscopic molecular structure of a substance can significantly affects its macroscopic technical properties. The bitumen’s molecular weight also affects its macroscopic technical properties. Normally, the higher the average molecular weight, the worse the low-temperature performance. A previous study has shown that the chromatogram of 70# bitumen can be divided into three parts: RT < 16.7 min, 16.7 < RT < 18.8 min, and RT > 18.8 min. These three parts correspond to the large molecular size (LMS), medium molecular size (MMS), and small molecular size (SMS), respectively. Their number-average molecular weight and weight-average molecular weight are each ~434 Da. The chromatogram of BRA is shown in Figure 2. The molecular weight of BRA is much lower than that of the 70# bitumen, with a number-average molecular weight of 500 Da, and a weight-average molecular weight of 512 Da.

The chemical composition of bitumen can be changed under the action of heating, oxygen, and ultraviolet light [28,29]. After aging, the molecules of bitumen tend to increase, and the light components of bitumen (saturates and aromatics) convert to heavy components (resins and asphaltenes) [30]. The changes in the chemical components in bitumen during aging damage the component balance of bitumen, resulting in an attenuation of the macroscopic physical and rheological properties of the bitumen. For instance, with respect to physical properties, the softening point and viscosity of bitumen increase, while the penetration and ductility decrease; in terms of the rheological properties, the complex modulus of bitumen increases, while the phase angle decreases. The molecular weight of BRA is similar to that of the light components of bitumen; it can increase the contents of the light components (saturates and aromatics) of bitumen and reduce the relative contents of resins and asphaltenes. Therefore, the BRA can rebalance the chemical components of the aged bitumen by decreasing the relative content of large molecules and heavy components.

#### 3.1.2. Chemical Structure of BRA-Rejuvenated RAP

**Figure 3:**
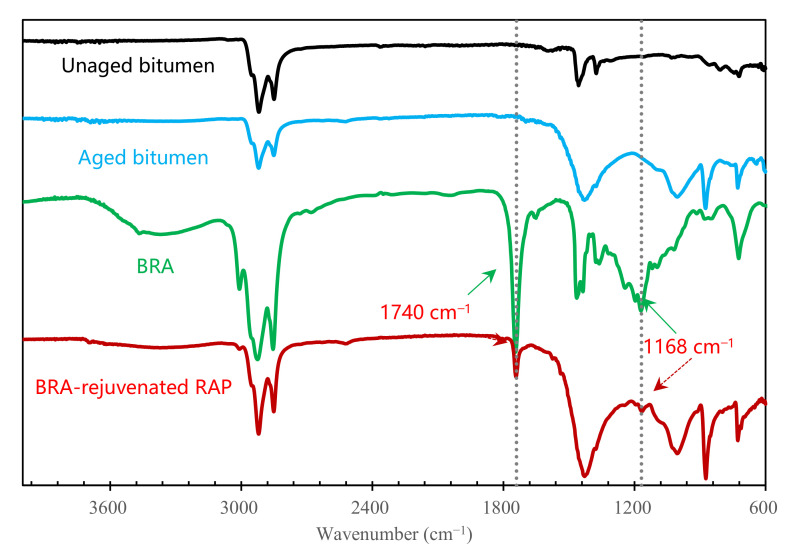
FTIR spectra of BRA and bituminous binders.

FTIR spectroscopy was used to investigate the chemical structure of the bitumen, as shown in Figure 3, where the absorption bands at around 1169 cm^−1^ and 1740 cm^−1^ belong to the characteristic absorption bands of BRA, which can only be found in the FTIR spectra of BRA and BRA-rejuvenated RAP; they cannot be found in the FTIR spectra of unaged bitumen or aged bitumen without BRA, showing that in the BRA-rejuvenated RAP, the BRA was successfully mixed with the aged bitumen. In addition, the FTIR spectrum of the BRA-rejuvenated RAP does not show any absorption band at a new wavenumber, but only a simple superposition of the bitumen and BRA absorption bands, indicating that no chemical reaction between the aged bitumen in the RAP and BRA occurred during the blending process of BRA and RAP. The mixing process of RAP and BRA is a physical process; it takes place mainly due to the penetration and diffusion of BRA into the aged bitumen, after which the BRA can gradually rebalance the chemical components and restore the performance of the aged bitumen covering the surface of the RAP.

### 3.2. Design and Optimisation of BRA-Rejuvenated RAP

#### 3.2.1. Effect of BRA Dosage on the Mechanical Properties of Bituminous Mixture

**Figure 4:**
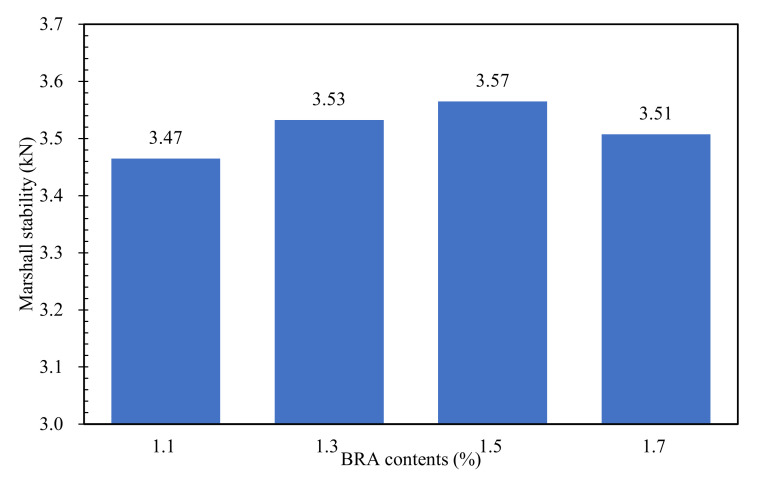
Effects of BRA dosages on the mechanical properties of BRA-rejuvenated RAP.

The early-stage mechanical properties of BRA-rejuvenated RAP were evaluated by the Marshall strength; the results are shown in Figure 4. As shown in Figure 4, when the BRA dosage was lower than 1.5%, the Marshall stability of the BRA-rejuvenated RAP increased with the increase in the BRA dosage; when the BRA content was higher than 1.5%, the Marshall stability of the BRA-rejuvenated RAP decreased with increasing BRA content. The mixture with 1.5% BRA had the highest Marshall stability, indicating that the BRA-rejuvenated RAP with 1.5% BRA had the best mechanical properties. This is because, on the one hand, BRA can activate the aged bitumen and rebalance its components, while on the other hand, the BRA can soften the aged bitumen, and offer the workability of RAP; the softening effect of BRA on the aged bitumen is also good for the compaction of BRA-rejuvenated RAP. When the BRA dosage is higher than 1.5%, the softening and viscosity reduction effects of BRA on the aged bitumen are dominant, being more obvious than the benefits in terms of workability, and decreasing the strength of the BRA-rejuvenated RAP. Thus, 1.5% BRA was suggested to be the optimal dosage for the rejuvenation of RAP.

#### 3.2.2. Effects of Low Dosages of New Bitumen on the Mechanical Properties of BRA-Rejuvenated RAP

**Figure 5:**
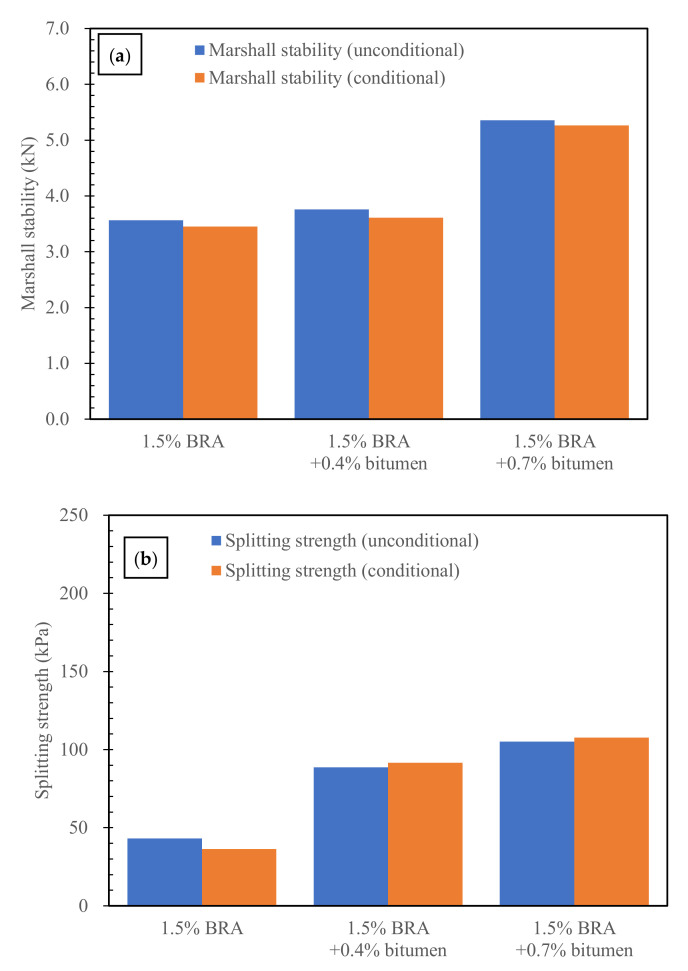
Effects of new bitumen on the mechanical properties of BRA-rejuvenated RAP: (**a**) Marshall stability; (**b**) splitting strength.

The results of the Marshall stability and splitting strength of BRA-rejuvenated RAP with low dosages of new bitumen are shown in Figure 5. As shown in Figure 5, the Marshall stability of BRA-rejuvenated RAP can satisfy the requirement of cold-recycled RAP (>3 kN) in the “Technical Specifications for Highway Asphalt Pavement Recycling” (JTG/T 5521—2019). After adding 0.7% new bitumen, the unconditional and conditional Marshall stability of BRA-rejuvenated RAP increased by 50.2% and 52.5%, respectively. Meanwhile, the unconditional and conditional splitting strength each increased by 195.5%. The results indicate that the conditioned and unconditioned Marshall stability and splitting strength of the BRA-rejuvenated RAP can be significantly increased by the addition of low dosages of new bitumen, and the improvement in mechanical properties was more significant with higher content of new bitumen.

#### 3.2.3. Effects of Low Dosages of Epoxy Resin on the Mechanical Properties of BRA-Rejuvenated RAP

**Figure 6:**
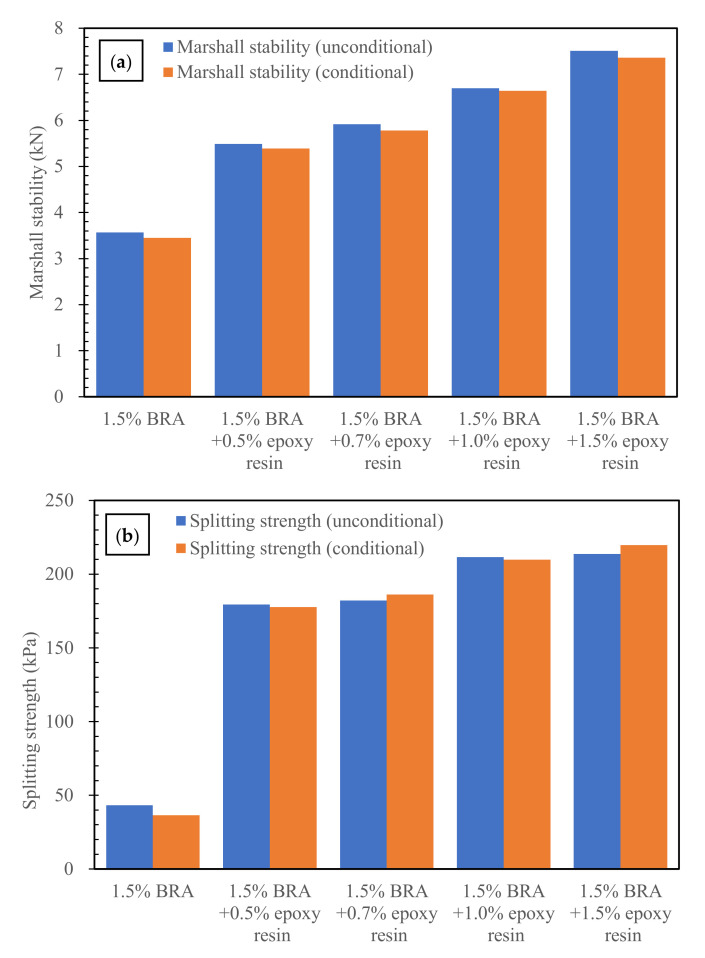
Effects of low dosages of epoxy resin on the mechanical properties of BRA-rejuvenated RAP: (**a**) Marshall stability; (**b**) splitting strength.

Figure 6 shows the results of the mechanical properties of the BRA-rejuvenated RAP with different dosages of epoxy resin. As shown in Figure 6, the epoxy resin can significantly improve the mechanical properties of BRA-rejuvenated RAP, and the improvement increases gradually with the increase in the epoxy resin dosage. The 0.5%, 0.7%, 1.0%, and 1.5% epoxy resin increased the unconditional Marshall stability by 54.0%, 66.0%, 87.8%, and 110.7%, respectively, and increased the unconditional splitting strength by 315.5%, 321.8%, 389.8%, and 394.4%, respectively. Compared with the same content of new bitumen, the beneficial effects of the epoxy resin on the mechanical properties of the BRA-rejuvenated RAP are more obvious. The reason for this is that, after curing, the epoxy resin has a high-strength and high-elasticity effect, and acts as a skeleton stiffener in the BRA-rejuvenated asphalt concrete, resulting in improvement of the early-stage mechanical properties of the recycled RAP.

Taking the above results of the mechanical properties of the RAP into consideration, the epoxy resin and BRA play different roles in the mixture. Due to the low molecular weight of BRA, the function of BRA is to soften the aged bitumen and decrease its viscosity. Another main function of BRA is to rebalance the chemical components of aged bitumen covering the surface of RAP, and to restore the technical performance of aged bitumen. Therefore, the RAP can be compacted at room temperature. The results show that all of the RAP samples could be compacted without heating. However, due to the low viscosity of bitumen, after curing, the mechanical strength of the RAP mixtures was not high. In order to enhance the mechanical strength of BRA-rejuvenated RAP, the epoxy resin was added to the mixture as a kind of reinforcing agent. After being mixed evenly in the mixture, the epoxy resin acted as a skeleton reinforcement, significantly improving the strength of BRA-rejuvenated RAP.

### 3.3. Road Performance of BRA-Rejuvenated RAP

#### 3.3.1. Water Damage Resistance of BRA-Rejuvenated RAP

The water damage resistance of BRA-rejuvenated RAP was investigated by using the water-immersed Marshall test and the freeze–thaw indirect tensile strength test [31]. The water-immersed residual strength (IRS) and freeze–thaw indirect tensile strength ratio (TSR) can be used to evaluate the water damage resistance of BRA-rejuvenated RAP [32]; the results are shown in Table 5 and Table 6. According to the “Technical Specifications for Construction of Highway Asphalt Pavement” (JTG F40-2004) and “Technical Specifications for Highway Asphalt Pavement Recycling” (JTG/T 5521—2019) in China, in order to satisfy the water damage resistance requirement, the IRS and TSR values of hot-mixed bituminous concrete should be no less than 80% and 75%, respectively. As shown in Table 5 and Table 6, the IRS and TSR values of BRA-rejuvenated RAP were 96.8% and 84.4%, respectively. These values are even higher than the water damage resistance requirements of hot-mixed bituminous concrete; therefore, the BRA-rejuvenated RAP has a good water damage resistance. In addition, the IRS values of BRA-rejuvenated RAP with low dosages of new bitumen or epoxy resin generally increase, and eventually approach 100%. With the addition of low dosages of new bitumen or epoxy resin, the TSR values of BRA-rejuvenated RAP increase significantly—0.7% new bitumen and 1.5% epoxy resin improve the TSR values of BRA-rejuvenated RAP by 18.0% and 12.8%, respectively. The results indicate that low dosages of new bitumen or epoxy resin can further improve the water damage resistance of BRA-rejuvenated RAP.

#### 3.3.2. Low-Temperature Cracking Resistance of BRA-Rejuvenated RAP

**Figure 7:**
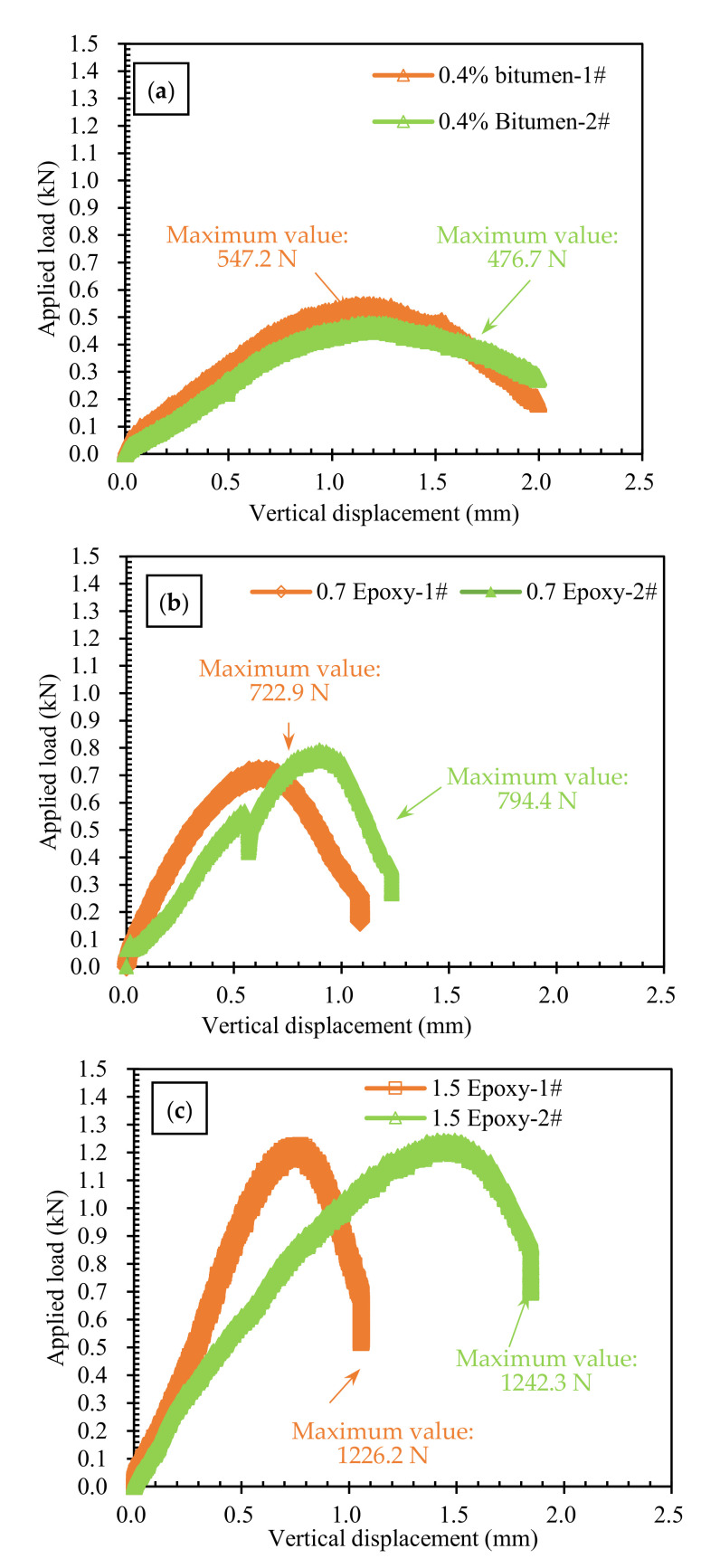
Displacement–load curve of −10 °C SCB test: (**a**) 0.4% bitumen; (**b**) 0.7% epoxy resin; (**c**) 1.5% epoxy resin.

The −10 °C SCB test was conducted to investigate the low-temperature cracking resistance of BRA-rejuvenated RAP, and the results are shown in Figure 7 and Table 7. From Figure 7 and Table 7, we can see that the maximum loadings of the BRA-rejuvenated RAP with 0.4% new bitumen, 0.7% epoxy resin, and 1.5% epoxy resin were 511.9 N, 758.6 N, and 1234.2 N, respectively, indicating that the 1.5%-epoxy-resin-reinforced BRA-rejuvenated RAP has the maximum bending and tensile strength. The differences in the the fracture work and fracture energy of BRA-rejuvenated RAP with 0.4% new bitumen and 0.7% epoxy resin was not significant; compared with them, the 1.5%-epoxy-resin-reinforced BRA-rejuvenated RAP showed an increase of 78.3% and 84.5%, respectively, indicating that the low-temperature performance of the 0.4%-new-bitumen- and 0.7%-epoxy-resin-reinforced RAP was essentially the same, while the 1.5%-epoxy-resin-reinforced RAP showed a significant increase.

#### 3.3.3. Fatigue Resistance of BRA-Rejuvenated RAP

**Figure 8:**
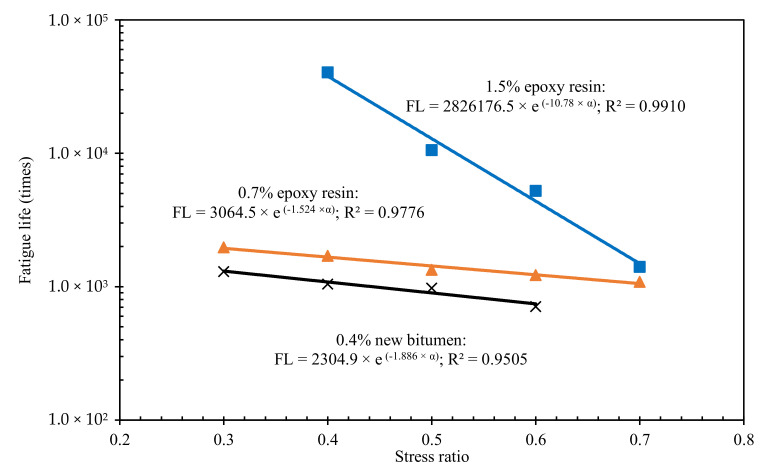
Fatigue life of the BRA-rejuvenated RAP.

Figure 8 shows the fatigue life results of BRA-rejuvenated RAP under different stress ratio conditions. As shown in Figure 8, compared with the 0.4%-new-bitumen-reinforced BRA-rejuvenated RAP, the fatigue resistance of the BRA-rejuvenated RAP was significantly improved by the addition of 0.7% and 1.5% epoxy resin. At a stress ratio of 0.4, the fatigue life of the 0.7%- and 1.5%-epoxy-resin-reinforced BRA-rejuvenated RAP was 1.6 times and 38.7 times that of BRA-rejuvenated RAP with 0.4% new bitumen, respectively. This indicates that the 0.7% and 1.5% epoxy resin are much more effective in improving the fatigue resistance of the BRA-rejuvenated RAP than the 0.4% new bitumen. The fatigue life of 0.7%-epoxy-resin-reinforced BRA-rejuvenated RAP is the least sensitive to load, which is beneficial to the fatigue performance of the road under large load adjustment. In addition, the fatigue curves can observe a change in the behaviour of epoxy-resin-reinforced BRA-rejuvenated RAP relative to the other two curves. The 1.5%-epoxy-resin-reinforced BRA-rejuvenated RAP has the highest sensitivity to stress ratio. The reason for this is that the mechanical strength of BRA-rejuvenated RAP is significantly enhanced by the 1.5% epoxy resin, and it is stiffer than the other two mixtures with 0% and 0.7% epoxy resin. The stiffness effect of 1.5% epoxy resin exerts a more obvious effect on the fatigue behaviour of BRA-rejuvenated BRA, but at the 0.7 stress ratio, it is still greater than 0.7%-epoxy-resin-reinforced BRA-rejuvenated RAP.

#### 3.3.4. High-Temperature Rutting Resistance of BRA-Rejuvenated RAP

**Figure 9:**
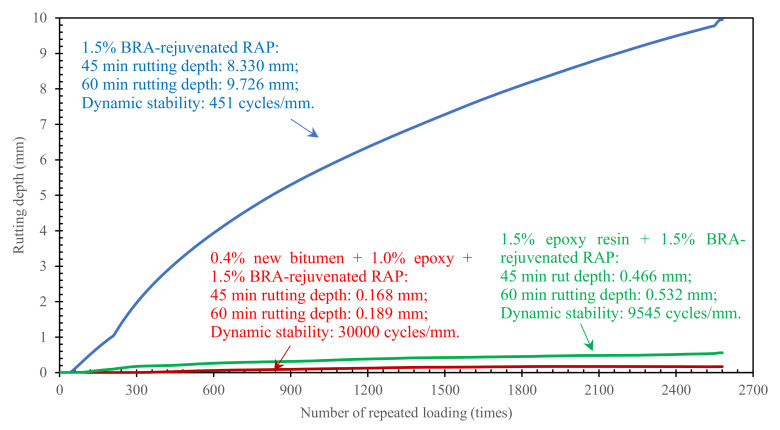
Rutting depth of BRA-rejuvenated RAP.

The lack of high-temperature rutting resistance is a typical problem for conventional cold-recycled and cold-mixed asphalt mixtures [33]. The high-temperature rutting resistance of BRA-rejuvenated RAP was investigated using the wheel tracking test, and the results are shown in Figure 9. The dynamic stability of the 1.5% BRA-rejuvenated RAP was only 451 cycles/mm, while after the addition of 1.5% epoxy resin and the combined addition of 0.4% new bitumen + 1.0% epoxy resin, the dynamic stability increased to 9545 cycles/mm and 30,000 cycles/mm, respectively. It can be seen that the rutting resistance of the BRA-rejuvenated RAP improved significantly with the addition of low doses of new bitumen and epoxy resin. The reason for this is that, after mixing and rejuvenation of the RAP with 1.5% BRA, there is still a very small amount of aggregate surface that is not covered with bitumen, and after compaction, this type of aggregate becomes the weak point in the BRA-rejuvenated bituminous concrete, resulting in insufficient rutting resistance. The addition of low doses of new bitumen enables the new asphalt to further coat the exposed aggregates and reduce the number of weak points in the bituminous concrete. Based on this, with the composite addition of epoxy resin, the epoxy resin acts as a skeleton reinforcement; therefore, the high-temperature rutting resistance of BRA-rejuvenated RAP can be improved significantly.

## 4. Conclusions

The chemical structure and molecular weight distribution of BRA and bitumen were investigated by FTIR and GPC to reveal the regeneration mechanism of BRA-rejuvenated RAP. In addition, the mechanical and road properties of BRA-rejuvenated RAP were investigated, and technologies were proposed to improve the road properties of BRA-rejuvenated RAP. The main conclusions are as follows:

(1) The molecular weight of BRA is similar to that of the light components of bitumen; it can increase the contents of light components and reduce the contents of heavy components of aged bitumen. The mixing process of RAP and BRA is a physical process; it mainly takes place due to the penetration and diffusion of BRA into the aged bitumen, after which the BRA can gradually rebalance the chemical components and restore the performance of the aged bitumen covering the surface of the RAP;

(2) The mixture with 1.5% BRA has the highest Marshall stability, indicating that the bituminous mixture has the best cohesive and mechanical properties. The early-stage mechanical properties (Marshall stability and splitting strength) of BRA-rejuvenated RAP can be significantly improved by the addition of low dosages of new bitumen (0.4%) and epoxy resin (0.5–1.5%);

(3) The IRS and TSR values of BRA-rejuvenated RAP are even higher than the water damage resistance requirements of HMA (no less than 80% and 75%, respectively), indicating that the BRA-rejuvenated RAP has good water damage resistance. The 0.4%-new-bitumen- and 0.7%-epoxy-resin-reinforced BRA-rejuvenated RAP had essentially the same low-temperature cracking resistance, while 1.5% epoxy resin reinforcement was significantly better. In comparison, the 0.7% and 1.5% epoxy resin reinforcements were much more effective in improving the fatigue resistance of the BRA-rejuvenated RAP than the 0.4% new bitumen. The rutting resistance of the BRA-rejuvenated RAP can be significantly improved by the addition of low dosages of new bitumen and epoxy resin;

(4) The epoxy resin and BRA play different roles in the BRA-rejuvenated RAP. Due to the low molecular weight of BRA, the function of BRA is to soften the aged bitumen and decrease its viscosity. Another main function of BRA is to rebalance the chemical components of aged bitumen covering the surface of RAP, and to restore the technical performance of aged bitumen. Therefore, the RAP can be compacted without heating; however, due to the low viscosity of bitumen, after curing, the mechanical strength of the RAP mixture is not high. The epoxy resin added to the mixture acts as a kind of reinforcing agent, and forms a skeleton that can significantly improve the strength of the BRA-rejuvenated RAP;

(5) It is feasible to use BRA as a regenerating agent to achieve 100% regeneration of RAP. In addition, the addition of low dosages of new bitumen or epoxy resin can further improve the road performance of BRA-rejuvenated RAP.

## Figures and Tables

**Table 1:** Technical parameters of BRA.

Technical Parameters	Units	Results	Experimental Methods
25 °C viscosity	cP	15	ASTM D4402
60 °C viscosity	cP	6	ASTM D4402
Specific gravity at 25 °C	-	0.90	ASTM D1475
Flash point	°C	>220	ASTM D92

**Table 2:** Particle size distribution of each RAP class.

Sieve Size (mm)	Passing Ratio (%) of Each RAP Aggregate			
	10–15 mm	5–10 mm	3–5 mm	0–3 mm
16	100	100	100	100
13.2	86.9	100	100	100
9.5	46.1	100	100	100
4.75	22.7	33.4	100	100
2.36	12.2	13.6	26.6	99.0
1.18	9.2	10.8	15.8	76.0
0.6	7.4	9.1	13.6	54.5
0.3	6.3	7.8	12.0	42.9
0.15	5.5	6.9	10.7	30.4
0.075	3.1	3.5	6.9	13.9

**Table 3:** Bitumen contents of different sizes of RAP aggregates.

**RAP Aggregate**	10–15 mm	5–10 mm	3–5 mm	0–3 mm
**Bitumen Content (%)**	3.1	3.5	3.6	7.5

**Table 4:** The dosages of new bitumen and epoxy resin.

Enhancement Additives	Dosages of Additives (%)				
New bitumen (%)	0	0.4	0.7	-	-
Epoxy resins (%)	0	0.5	0.7	1.0	1.5

**Table 5:** Effects of new bitumen on the water damage resistance of BRA-rejuvenated RAP.

**Dosage of New Bitumen (%)**	0	0.4	0.7
**IRS (%)**	96.8	96.0	98.3
**TSR (%)**	84.4	103.4	102.4

**Table 6:** Effects of epoxy resin on the water damage resistance of BRA-rejuvenated RAP.

**Dosage of Epoxy Resin (%)**	0	0.5	0.7	1.0	1.5
**IRS (%)**	96.8	98.2	97.7	99.2	98.0
**TSR (%)**	84.4	99.0	97.9	99.2	97.2

**Table 7:** Average fracture energy of −10 °C SCB test.

Additives	Dosages (%)	Maximum Loading (N)	Fracture Work (J)	Fracture Energy (J/m^2^)
New bitumen	0.4	511.9	6.0	2101.2
Epoxy resin	0.7	758.6	5.8	2045.5
Epoxy resin	1.5	1234.2	10.7	3739.9

## Data Availability

All the data is available within the manuscript.
